# Primary Bilateral Non-Hodgkin's Lymphoma of the Adrenal Gland Presenting as Incidental Adrenal Masses

**DOI:** 10.1155/2015/620381

**Published:** 2015-11-22

**Authors:** Christopher Rizzo, David James Camilleri, Alexandra Betts, Andre' Gatt, Stephen Fava

**Affiliations:** ^1^Diabetes and Endocrine Centre, Mater Dei Hospital, Msida MSD 2090, Malta; ^2^Department of Medicine, University of Malta Medical School, Msida MSD 2080, Malta; ^3^Department of Haemato-Oncology, Mater Dei Hospital, Msida MSD 2090, Malta; ^4^Histopathology Section, Pathology Department, Mater Dei Hospital, Msida MSD 2090, Malta; ^5^Medical Imaging Department, Mater Dei Hospital, Msida MSD 2090, Malta

## Abstract

Although lymphoma may occasionally involve the adrenal glands as part of a generalized disease process, primary adrenal lymphoma (PAL) is a rare disease. We present a case of a 62-year-old woman with a history of mild/moderate hereditary spherocytosis with a well-compensated baseline haemoglobin, who presented with rapidly progressive symptomatic anaemia. During the diagnostic workup, imaging revealed bilateral large adrenal masses and she was later diagnosed with diffuse large B-cell non-Hodgkin's lymphoma (DLBCL), with the adrenal glands being the dominant site of the disease. The patient was started on systemic chemotherapy, but her disease progressed with neurological involvement which responded to second-line therapy. Her adrenal disease however was refractory to further therapy.

## 1. Introduction

Malignant lymphoma arising primarily in the endocrine organs is rare, accounting for less than 8% of extranodal presentations, and among the endocrine organs, the thyroid gland is most commonly involved [1].

PAL is a rare condition and may present as bilateral adrenal masses which may be rapidly growing. Patients usually do not have disease elsewhere and this makes the diagnosis more difficult.

Symptoms of the disease are variable and patients are most commonly older males. Presenting clinical features include pyrexia, lumbar pain, and/or symptoms of adrenal insufficiency. It may also be diagnosed incidentally on abdominal imaging.

## 2. Case Presentation

A 62-year-old lady known to suffer from hereditary spherocytosis (HS) was referred to hospital with progressively worsening symptomatic anaemia (Hb 7.7 g/dL), over a three-week period.

She was afebrile and had a BP of 140/90 mmHg. Examination of her cardiovascular and respiratory systems was unremarkable. There was no palpable lymphadenopathy, but splenomegaly, in keeping with her underlying diagnosis of HS, was detected. There was no history of iatrogenic immunosuppression or autoimmune disease.

A blood picture revealed spherocytosis and reticulocytosis. An erythrocyte sedimentation rate (ESR) was raised at 100 mm/h with a raised CRP of 15 mg/L.

Her urea and electrolytes were within normal limits. Bilirubin was elevated at 22 umol/L (1.72–17.7), with normal liver enzymes. Renal function, serum calcium, phosphate, and albumin were normal. A direct Coombs test was negative.

Serum ferritin was elevated at 487 ng/mL (10–291) with normal serum iron and TIBC levels. Serum folate and vitamin B12 levels were normal.

**Figure 1:**
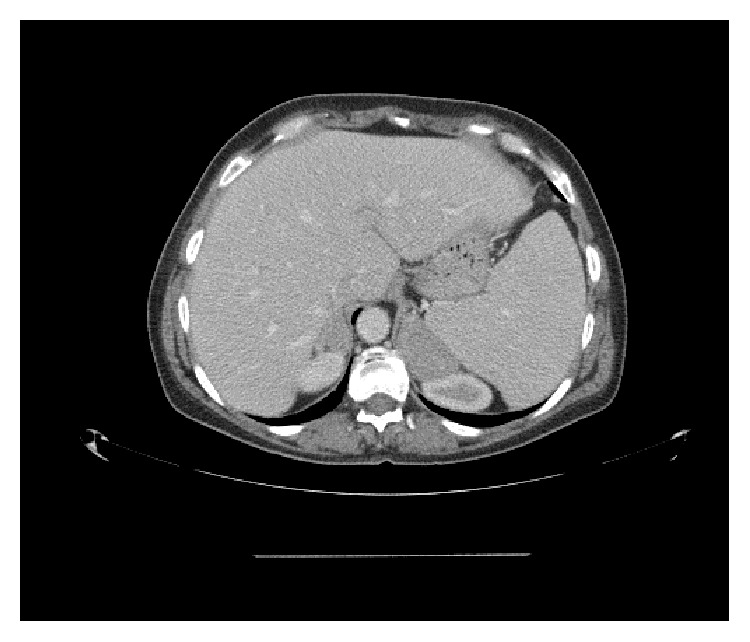
CT of the abdomen showing bilateral, homogenous adrenal gland enlargement.

As part of her initial workup a CT, neck and trunk, was performed, which revealed bilateral, marked, homogenous adrenal gland enlargement (Figure 1), measuring 3.2 × 1.4 cm on the right and 5.2 × 3.0 cm on the left, with concomitant splenomegaly.

Serum and 24-hour urinary catecholamines and metanephrines were not elevated. 24-hour urine cortisol levels were within normal limits: 635 nmol/24 hours (57–806). Serum cortisol at 09:00 h was 542 nmol/L (119–618).

Within a few weeks, whilst awaiting the adrenal biopsy, our patient presented to hospital with nausea, vomiting, and generalized aches and pains and new onset of submandibular lymphadenopathy.

At this point investigations revealed a white blood cell count of 4 × 10^9^/L, haemoglobin 8.6 g/dL, and a platelet count of 197 × 10^9^/L with reticulocytosis. Urea and electrolytes and creatinine were again within normal limits. Liver function tests revealed mild unconjugated hyperbilirubinemia of 22.0 umol/L and a normal albumin and INR. The liver enzymes were mildly deranged, alkaline phosphatase (ALP): 290 U/L (40–104), alanine transaminase (ALT): 68 U/L (5–33), and gamma-glutamyl transferase (GGT): 104 U/L (5–36). Iron profile revealed a raised ferritin with normal iron levels, as at initial presentation. Serum lactate dehydrogenase (LDH) was elevated at 488 U/L (135–250). Serum protein electrophoresis revealed elevated gamma globulin levels but no monoclonal band. Tumour markers (carcinoembryonic antigen and CA19-9) were negative and ESR was still raised at 114 mm/h (18–22), with a mildly raised CRP of 16 mg/L (0–10).

**Figure 2:**
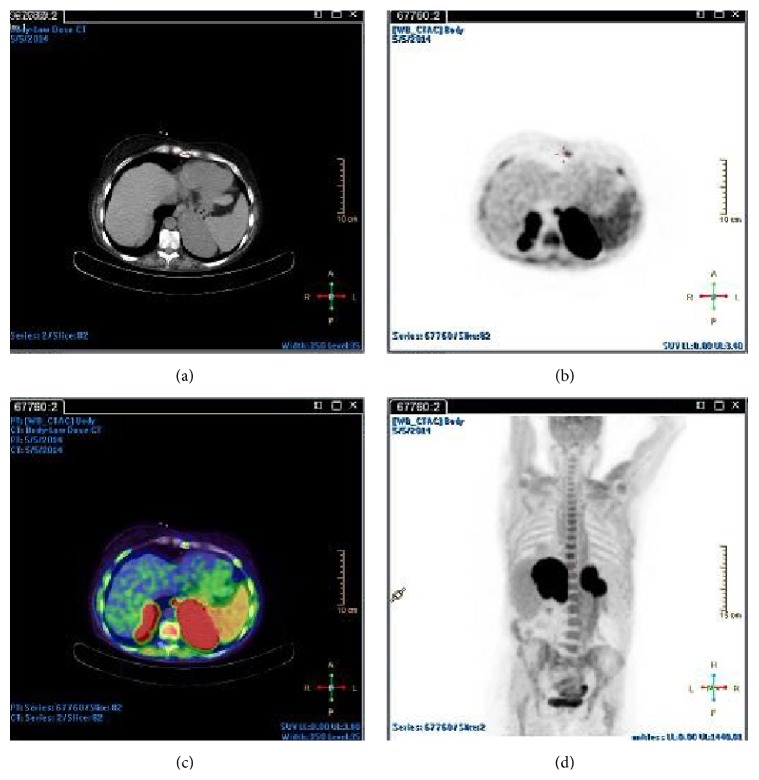
Fluorodeoxyglucose positron emission tomography demonstrating extensive uptake in both adrenal glands.

**Figure 3:**
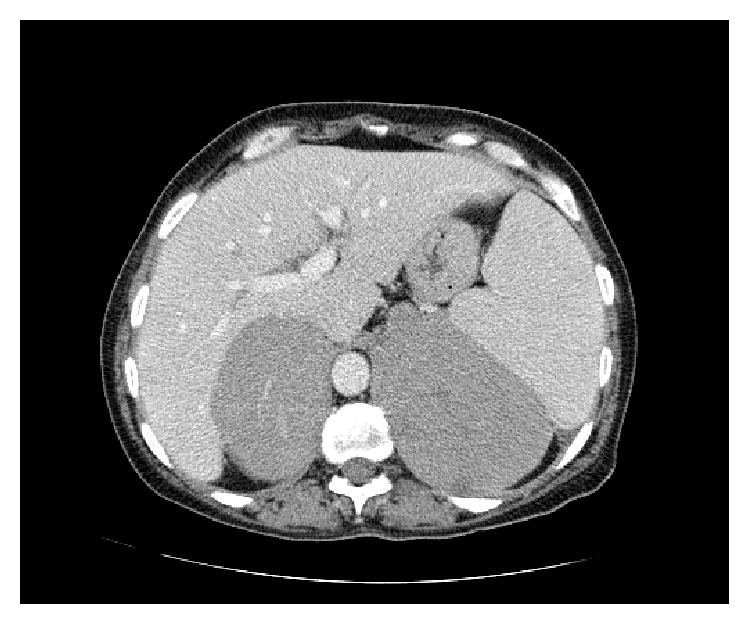
Spiral 64-slice CT scan obtained following intravenous contrast (100 mL Iohexol) in portovenous phase demonstrates marked, homogenous bilateral adrenal gland enlargement with no evidence of disease elsewhere.

Flouorordeoxyglucose positron emission tomography (FDG PET) CT at this point revealed avid tracer uptake in several pathologically enlarged lymph node groups above and below the diaphragm, including both adrenal masses (Figure 2), with the latter having more than doubled in size from the CT at presentation and which now measured 7 × 9 cm on the right and 8 × 12 cm on the left (Figure 3).

An excision biopsy of an axillary lymph node was performed. The axillary lymph node was biopsied in preference to an adrenal biopsy for logistical reasons.

**Figure 4:**
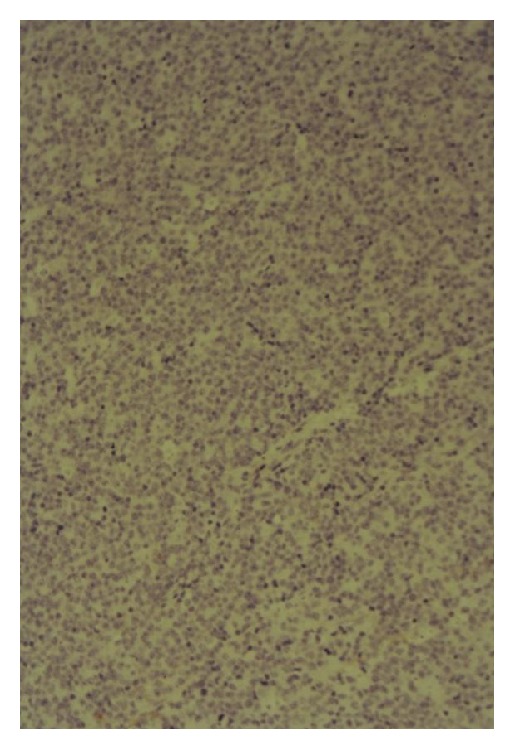
Lymph node biopsy immunohistochemical staining showing negative CD10 expression.

**Figure 5:**
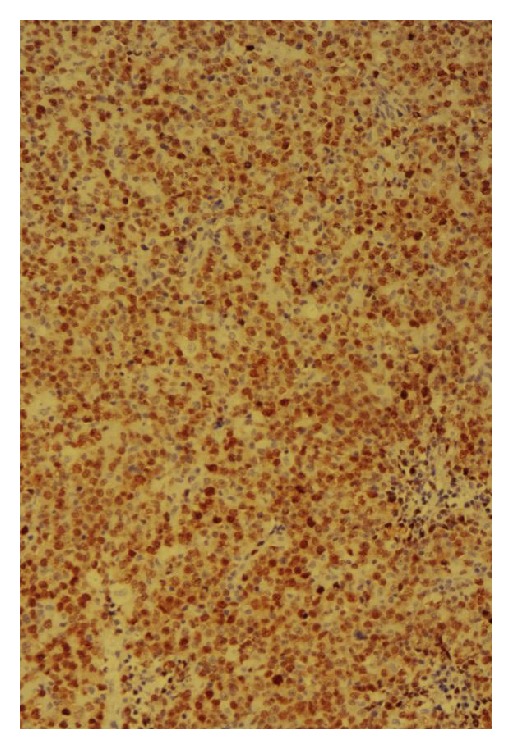
Lymph node biopsy immunohistochemical staining showing MUM1 expression.

Histology confirmed diffuse large B-cell lymphoma, activated B-cell phenotype (Figures 4 and 5).

Her international prognostic index (IPI) score [2] was calculated at 4/5, based on her age (>60; +1), stage (IV; +1), LDH (raised; +1), extranodal sites (×2; +1), and performance status (1; 0). This put her in the high-risk category with a median 5-year overall survival of ~40%. Her bone marrow was not involved at diagnosis.

## 3. Treatment

R-CHOP chemotherapy (rituximab, cyclophosphamide, doxorubicin, vincristine, and prednisolone) was initiated, but despite clinical resolution of palpable lymphadenopathy after 2 cycles, she developed bilateral lower limb weakness and paraesthesia.

MRI of the brain and spine did not reveal any evidence of leptomeningeal disease or cord compression, but lumbar puncture revealed a markedly raised cerebrospinal fluid (CSF) protein 3054 mg/L (150–600), with numerous blast-like cells, compatible with involvement of CSF by lymphoma.

She was started on HyperCVAD/MA-R (hyperfractionated cyclophosphamide, vincristine, doxorubicin, and dexamethasone alternating with high-dose methotrexate, cytosine arabinoside, and rituximab), a regimen which includes drugs which cross the blood-brain barrier, together with intrathecal chemotherapy.

**Figure 6:**
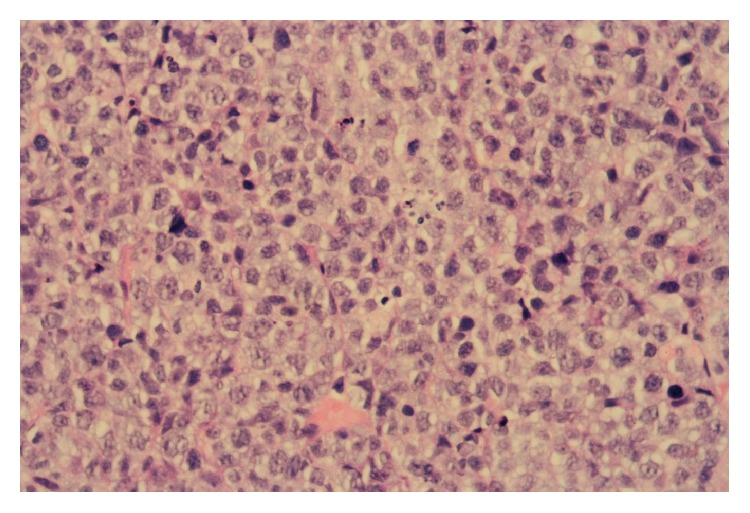
H&E stain showing large atypical lymphoid cells with apoptotic debris and mitotic figures (high power magnification).

**Figure 7:**
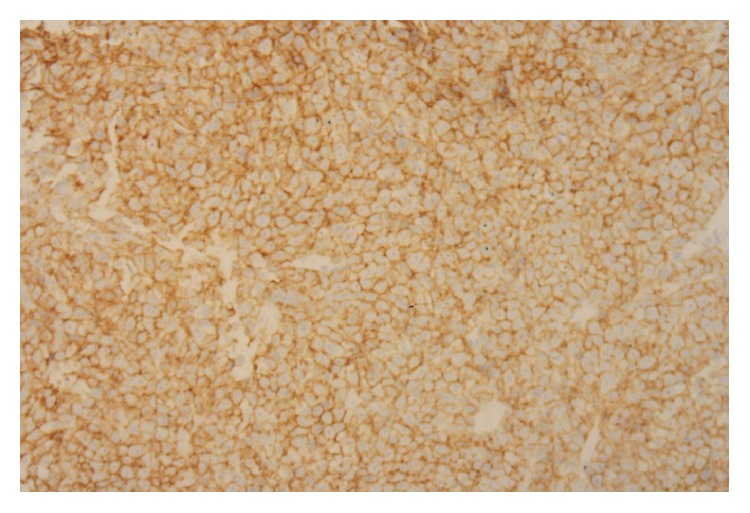
Adrenal gland biopsy showing lymphoid cell positivity for B-cell marker CD20.

**Figure 8:**
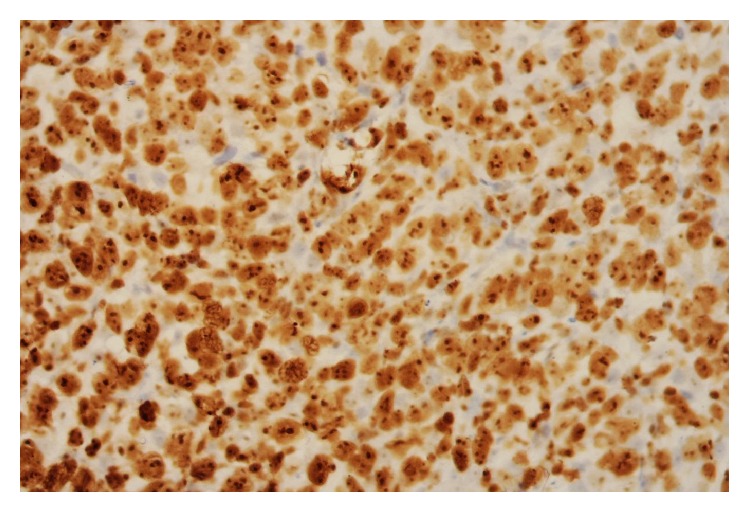
Ki67 immunoperoxidase stain showing positive nuclear staining of the lymphoma cells (Ki67 index ~ 85%), indicating a high proliferative rate.

**Figure 9:**
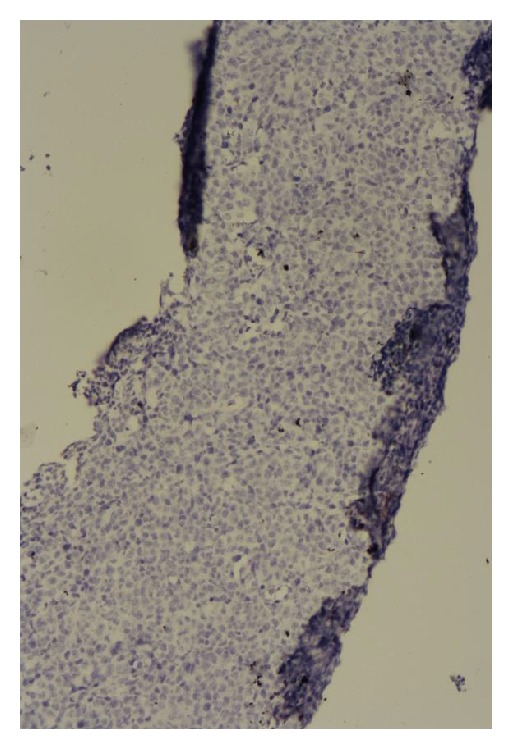
Adrenal biopsy immunohistochemical staining showing negative CD10 expression.

**Figure 10:**
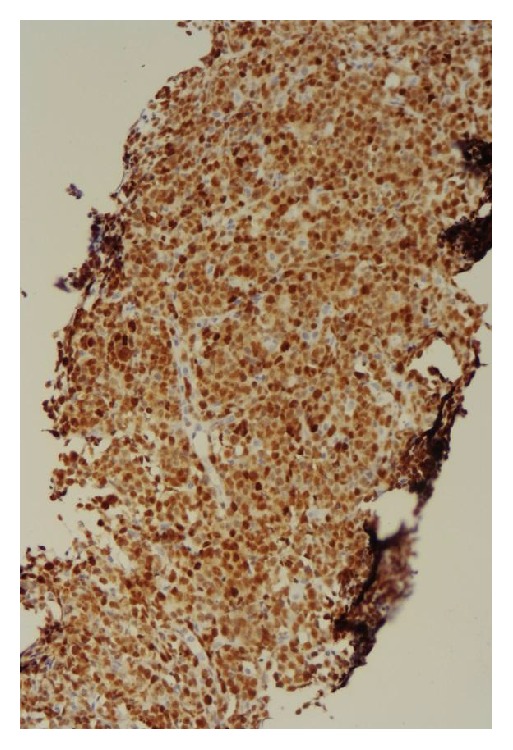
Adrenal biopsy immunohistochemical staining showing positive MUM1 expression.

A repeat FDG PET-CT after 4 cycles of this regimen revealed resolution of disease in all lymph node groups, a clear CSF, but persistent disease in both adrenals, which did not meet criteria for partial remission by radiological criteria. Biopsy of the adrenals at this stage was performed to exclude a second concomitant pathology. The adrenal biopsy revealed DLBCL of the activated B-cell phenotype (Figures 6, 7, 8, 9, and 10).

At this point, in view of persistent disease, a decision was made to change to third-line chemotherapy and work up the patient for an autologous peripheral stem cell transplant.

The regimen chosen was R-GemCEBOM (rituximab, gemcitabine, cyclophosphamide, etoposide, vincristine, and methotrexate), which included intrathecal administration of further chemotherapy and drugs she had not been exposed to in previous regimens, namely, gemcitabine and etoposide.

This intensive weekly regimen was administered for the duration of 12 weeks with full supportive measures, but some delays were inevitable due to toxicity and neutropenic sepsis, with ITU transfer occurring on two occasions, with the added problem of hypoadrenalism due to adrenal infiltration.

Half-way through therapy, at 6 weeks, a restaging FDG PET-CT revealed some reduction in adrenal disease, but an end-of-treatment repeat scan showed further progression of the adrenal masses.

At this point it was decided to go for a palliative approach. Pulses of high-dose methylprednisolone together with adrenal irradiation and pain relief were instituted. The patient passed away 4 weeks following the institution of palliative care and 1 year following initial diagnosis.

## 4. Discussion

PAL is a rare disorder [3, 4] and more common in men from the sixth decade onwards [5]. The majority of cases involve both adrenal glands and bulky disease is more common [4].

The main clinical features are local symptoms such as lumbar pain or systemic symptoms such as fever and weight loss. Symptoms of adrenal insufficiency such as vomiting, fatigue, skin hyperpigmentation, and hypotension can also occur especially if there is extensive bilateral adrenal involvement [6, 7]. Uncommon presentations include autoimmune haemolytic anaemia [8], thrombocytopenia, decreased visual acuity resulting from choroidal metastases [9], hypercalcaemia [6], and concomitant involvement of other extranodal sites. The exact pathogenesis is still unknown, although association with autoimmune diseases and immunodeficiency states has been described [10]. Epstein-Barr virus (EBV) has been associated with the development of endemic, sporadic, and AIDS-associated Burkitt lymphoma. It has also been described in the pathobiology of lymphoma in the setting of immunosuppressive therapy, such as after organ transplantation, or in the setting of chronic low-dose methotrexate therapy, usually used in the treatment of rheumatic diseases [11, 12].

Diagnosis can also be made incidentally on abdominal imaging.

In the differential diagnosis of bilateral adrenal masses one must consider metastatic deposits, bilateral phaeochromocytoma, granulomatous disorders, and rarely adrenal myelolipoma and adrenocortical carcinoma.

CT scan will generally show homogenous masses with predominantly low density and slight to moderate enhancement [4, 13]. These features are however not pathognomonic.

The most common histology in PAL is diffuse large B-cell lymphoma, usually activated B-cell phenotype. T-cell and angiocentric large cell lymphoma type B are the exception [14].

Histological diagnosis is a gold standard in evaluation of lymphoma. Prognosis is usually poor and the median survival is usually less than 1 year [4, 15]. Poor prognostic factors include advanced age, large tumour size, bilateral involvement, high LDH levels, involvement of other organs, and initial presentation with adrenal insufficiency [2, 4]. The germinal centre subtype (GCB) seems to have a better prognosis than the non-GCB subtype [16]. CNS involvement worsens long-term prognosis and the addition of rituximab has significantly improved outcomes in PAL [13].

PAL usually does not have disease found elsewhere, but if it is present, it is more likely extranodal in nature. Sites for extranodal involvement for PAL are the CNS and the gastrointestinal tract, as well as other endocrine organs [3]. In PAL, disease may also relapse in the CNS and the risk of CNS relapse may be high [4].

The therapeutic modalities include combination chemotherapy and radiotherapy in order to prevent local recurrence. Surgical debulking is not recommended in view of high morbidity and mortality and recurrence of the disease elsewhere later [4].

In conclusion, this rare disease entity has to be considered in the differential diagnosis of endocrinologically silent adrenal masses, especially if bilateral or rapidly growing. The diagnosis is essentially histological and this highlights the importance of obtaining a tissue biopsy early on in the diagnostic workup of bilateral adrenal masses. PAL is an aggressive type of DLBCL, and prognosis is generally quite poor [13, 16]. The most appropriate therapeutic approach has not been identified, due to the lack of randomized controlled trials, with this being a rare disorder.

## 5. Learning Points/Take-Home Messages

1. Primary adrenal lymphomas are rare tumours.
2. PAL should be considered in the differential diagnosis of unilateral and bilateral adrenal masses.
3. Patients may present with clinical features of adrenal insufficiency.
